# ALPK2 acts as tumor promotor in development of bladder cancer through targeting DEPDC1A

**DOI:** 10.1038/s41419-021-03947-7

**Published:** 2021-07-01

**Authors:** Yuchen Wang, Jie Wu, Wenjie Luo, Hailiang Zhang, Guohai Shi, Yijun Shen, Yao Zhu, Chunguang Ma, Bo Dai, Dingwei Ye, Yiping Zhu

**Affiliations:** 1 Department of Urology, Fundan University Shanghai Cancer Center, No. 270 Dongan Road, 200032 Shanghai, People’s Republic of China; 2 Department of Oncology, Shanghai Medical College, Fudan University, No. 270 Dongan Road, 200032 Shanghai, People’s Republic of China

**Keywords:** Cancer models, Bladder cancer

## Abstract

Bladder cancer is one of the most common malignant tumors in the urinary system. The development and improvement of treatment efficiency require the deepening of the understanding of its molecular mechanism. This study investigated the role of ALPK2, which is rarely studied in malignant tumors, in the development of bladder cancer. Our results showed the upregulation of ALPK2 in bladder cancer, and data mining of TCGA database showed the association between ALPK2 and pathological parameters of patients with bladder cancer. In vitro and in vivo experiments demonstrated that knockdown of ALPK2 could inhibit bladder cancer development through regulating cell proliferation, cell apoptosis, and cell migration. Additionally, DEPDC1A is identified as a potential downstream of ALPK2 with direct interaction, whose overexpression/downregulation can inhibit/promote the malignant behavioral of bladder cancer cells. Moreover, the overexpression of DEPDC1A can rescue the inhibitory effects of ALPK2 knockdown on bladder cancer. In conclusion, ALPK2 exerts a cancer-promoting role in the development of bladder cancer by regulating DEPDC1A, which may become a promising target to improve the treatment strategy of bladder cancer.

## Introduction

Bladder cancer is one of the most prevalent types of cancer in the world, with 549,000 new cases of bladder cancer worldwide, and 199,900 dead cases of bladder cancer per year, which is still rapidly increasing [1–3]. Bladder cancer originates from the translational epithelium (urothelial epithelium) of the bladder, the most common type of which is urothelial carcinoma (>95%) [4]. Approximate 70–75% of patients are diagnosed with non-muscular invasive disease, while the remaining 25–30% present with infiltrating tumor cells in the musculature of the bladder wall. Despite radical cystectomy and pelvic lymph node dissection have good efficiency for treating non-muscle invasive bladder cancer, about 50% of patients relapse and worsen the disease due to disseminated micro-metastasis [5, 6]. The presence of muscle-invasive and advanced bladder cancer requires platinum-based chemotherapy and more systematic treatment, but the results are unsatisfactory [4, 7]. In recent years, molecular targets have been gradually developed for precision therapy, such as fibroblast growth factor receptor inhibitors, antibody-drug conjugates, and interference with long non-coding RNA, microRNA, and PARP1 as well as receptor signal pathways [8–12]. Therefore, further exploration of the molecular characteristics of bladder cancer is conducive to identify novel potential targets for bladder cancer [13, 14].

The *ALPK2* gene was initially found to possess a domain with pretty high similarity to the elongation factor 2 kinase catalytic domain [15], which is mapped to chromosome 18q21.31, as well as the distal end of a minimal region of loss of heterozygosity commonly observed in colorectal cancer [16, 17]. Furthermore, a comprehensive sequencing aiming to identify somatic mutations in potentially oncogenic kinases revealed *ALPK2*, as one of the mutated genes in human cancers such as ovarian cancer [18, 19]. Alpha protein kinase 2 (ALPK2) is a member of an atypical alpha protein kinase family including 6 alpha kinases: eukaryotic elongation factor 2 kinase (eEF2K), TRP ion channel proteins (TRPM6 and TRPM7) as well as lymphocyte alpha kinase (LAK or ALPK1), heart alpha kinase (HAK or ALPK2), and muscle alpha kinase (MAK or ALPK3) [20, 21]. Recently, ALPK2 was identified as a key regulator in cardiac development and an effective inhibitor of the Wnt/β-catenin signaling pathway, which could promote cardiogenesis in zebrafish and human pluripotent stem cells [22]. Although the biological function of ALPK2 and its role in malignant tumor has been studied to some extent, the understanding of ALPK2 is still far from enough. To the best of our knowledge, the relationship between ALPK2 and bladder cancer is rarely reported and still unclear.

In this study, ALPK2 is recognized to be abundantly expressed in bladder cancer, whose expression is higher in bladder cancer tissues than normal tissues. In addition, knockdown of ALPK2 can inhibit the development of bladder cancer in vivo and in vitro. Additionally, DEPDC1A is identified as a potential downstream of ALPK2, whose overexpression/downregulation can inhibit/promote the malignant behavioral of bladder cancer cells. Notably, overexpression of DEPDC1A can interfere with the inhibitory effects of ALPK2 knockdown on bladder cancer. Therefore, ALPK2 exerts a cancer-promoting role in the development of bladder cancer by regulating DEPDC1A, which may become a promising target to improve the treatment strategy of bladder cancer.

## Materials and methods

### Clinical specimens, cell culture, and antibodies

The formalin-fixed, paraffin-embedded tissue microarray of bladder cancer were purchased from Shanghai Outdo Biotech Company (Shanghai, China). Written informed consent and the characteristic information of all patients were obtained. The study was approved by Ethics committee of Fudan University. All tissues were fixed with formalin for 24 h and embedded with paraffin. Sections of 5 μm were cut and prepared.

Human bladder cancer cell lines J82 and RT4 cell lines were obtained from the BeNa Technology (Hangzhou, Zhejiang, China). T24 were purchased from American Type Culture Collection (ATCC, Manassas, VA, USA). Cell line EJ was obtained from Genechem (Shanghai, China). EJ, RT4, and T24 cell lines were cultured in RPMI-1640 medium (Gibco, Rockville, MD, USA) containing 10% FBS (Gibco, Rockville, MD, USA) at 37 °C with 5% CO_2_ and cell medium were exchanged every three days. J82 cells were maintained in 90% EME medium (Gibco, Rockville, MD, USA) supplemented with 10% FBS additive.

Antibodies used in our study were showed in Table S1.

### Immunohistochemistry analysis

Bladder cancer and non-tumor bladder tissue sections were dewaxed by dimethylbenzene and dehydrated by gradient alcohol. Endogenous peroxidase was blocked with 3% H_2_O_2_ and antigen retrieval was accomplished with boiling citric acid buffer. The sections were cooled naturally and washed. Then the sections were incubated with anti-ALPK2 at 4 °C overnight and continuingly incubated with the secondary antibody for 2 h at room temperature. Finally, the sections were stained with diaminobenzidine and exanimated with ImageScope_v11.2.0.780 and CaseViewer_2.0_RTM_v2.0.2.61392. Sections were classified into negative (0), positive (1–4), ++ positive (5–8), or + ++ positive (9–12), based on the sum of the staining intensity (varied from weak to strong) and staining extent scores, which graded as 0 (0%), 1 (1–25%), 2 (26–50%), 3 (51–75%), or 4 (76–100%). The high/low grouping was made based on the median of the IHC scores of all tissue samples.

### Plasmid construction, lentivirus infection, and transfection

Interfering sequences targeting ALPK2 or DEPDC1A were designed by Shanghai Bioscienceres (Shanghai, China) (5′-3′, ALPK2: GCGAAGACCTTGGCATTTATT; TGCTAATAATGAGTGCTTTCA. DEPDC1A: TGTTGAAGAAGTTTGGAGATA; AACGAGATGTATTCAGAACAA). cDNAs containing relevant targenting sequence was synthesized and double strand DNAs were prepared. DNAs were cloned into BR-V108 lentivirus vector according to the Fermentas T4 DNA Ligase instructions. Then BR-V108 lentivirus vector were transferred into Top 10 *E. coli* receptor cells (TIANGEN, Cat. #CB104-03). Positive clones were sequenced by PCR and qualified plasmids were extracted by EndoFree Maxi Plasmid Kit (TIANGEN, Beijing, China) for packaging.

Four hundred microliter 1 × 10^7^ TU/mL lentivirus were transfected into EJ and T24 cells using Lipofectamine 2000 transfection reagent (Thermo Fisher Scientific, Waltham, MA, USA). After cultured for 72 h, cell infection efficiency was evaluated by microscopic fluorescence.

### RNA extraction and RT-qPCR

Lentivirus transfected cells EJ, T24, J82, and RT4 were cultured for 72 h, and total RNA was extracted using TRIzol reagent (Sigma, St. Louis, MO, USA), respectively. Nanodrop 2000 spectrophotometer (Thermo Fisher Scientific, Waltham, MA, USA) was used to determine the quality of RNA according to the manufacturer’s instructions. Then 2.0 μg RNA sample was reverse transcribed to cDNA and quantitative real-time PCR was conducted with SYBR Green mastermixs Kit (Vazyme, Nangjing, Jiangsu, China) and 2^−ΔΔCt^ method was applied to evaluate the relative quantitative. Inner control of PCR was GAPDH. Primers used were showed in Table S2.

### Western blotting assay

Total protein was collected after T24 and EJ cells were lysed in ice-cold RIPA buffer (Millipore, Temecula, CA, USA), and the concentration was detected by BCA Protein Assay Kit (HyClone-Pierce, Logan, UT, USA). Then protein sample (20 µg) was separated by 10% SDS-PAGE (Invitrogen, Carlsbad, CA, USA) and were transferred onto PVDF membranes. The membranes were incubated with primary antibodies at 4 °C overnight, and continuingly incubated with the secondary antibody for 2 h at room temperature. Enhanced chemiluminescence (ECL) (Amersham, Chicago, IL, USA) was applied for the blots visualizing.

### Celigo cell counting assay

Seventy-two hours after the transfection, T24 cells were collected and seeded into a 96-well plate with a cell density of 2000 cells per well. Cells were further cultured in RPMI-1640 medium containing 10% FBS at 37 °C with 5% CO_2_ for 24 h. Cell counting was accomplished by Celigo image cytometer (Nexcelom Bioscience, Lawrence, MA, USA) at day 1, 2, 3, 4, 5, and the cell proliferation curve was drawn.

### Flow cytometry for apoptosis and cell cycle

Lentivirus transfected EJ and T24 cells were inoculated in 6-well plates with 2 mL per well (1 × 10^3^ cells/mL) in triplicate and further cultured for 5 days. Cells were collected, trypsinized and washed with 4 °C ice-cold D-Hanks. After centrifugation (1000 × *g*), cells were resuspended with binding buffer, then 10 μL Annexin V-APC (eBioscience, San Diego, CA, USA) was added for staining in dark. Apoptosis analysis was measured using FACSCalibur (BD Biosciences, San Jose, CA, USA).

For cell cycle detection, cells were stained by 5 μL Annexin V-APC (eBioscience, San Diego, CA, USA) in dark for 15 min. Then propidium iodide solution (PI, Sigma, St Louis, MO, USA) were added and cell cycle distribution was detected by FACSCalibur and observed by micropublisher (Olympus, Tokyo, Japan), and cells in G0-G1, S, and G2-M phase were counted and the percentage were compared.

### Wound healing assay

Lentivirus infected T24 cells were seeded onto a 96-well dish. After synchronization, a 96-wounding replicator (VP scientific, San Diego, CA, USA) was used to make scratch crossing the cell layer, and floating cells were washed away. RPMI-1640 medium with 0.5% FBS was added for culturing. Photographs were taken by a fluorescence microscope at 0, 8, and 24 h after scratching. Cell migration rate of each group was calculated.

### Transwell assay

Transwell assay was performed by Corning Transwell Kit (Corning, NT, USA). First, exponentially growing T24 cells were collected, trypsinized, counted and incubated in the upper chamber with 100 μL medium without FBS in a 24-well plate (5 × 10^4^ cells/well). Six hundred microliter medium supplemented with 30% FBS was added in the lower chamber. Cells were incubated for 24 h at 37 °C with 5% CO_2_ and non-metastatic cells were removed with a cotton swab. Cells were fixed by 4% formaldehyde and 400 µL Giemsa was added for staining and the migration ability of cells was analyzed.

### PrimeView human gene expression array

The detection of gene expression profile in T24 cells transfected with shALPK2 or shCtrl by RNA screening analysis was completed by Shanghai Bioscienceres, Co., Ltd. (Shanghai, China). Total RNA was extracted by the RNeasy kit (Sigma, St. Louis, MO, USA). Concentration and values of A260 and A280 of total RNA were determined by Nanodrop 2000 (Thremo Fisher Scientific, Waltham, MA, USA). RIN value was evaluated with Agilent 2100 and Agilent RNA 6000 Nano Kit (Agilent, Santa Clara, CA, USA). RNA sequencing was performed with Affymetrix human GeneChip PrimeView according to the manufacturer’s instruction and the outcomes were scanned by Affymetrix Scanner 3000 (Affymetrix, Santa Clara, CA, USA). Raw data statistical significance assessment was accomplished using a Welch *t*-test with Benjamini–Hochberg FDR (|Fold Change| ≥ 2.0 and FDR < 0.05 as significant). Significant difference analysis and functional analysis based on Ingenuity Pathway Analysis (IPA) (Qiagen, Hilden, Germany) was executed, and |*Z*-score| > 2 is considered meaningful.

### Human apoptosis antibody array

Detection of related genes in human apoptosis signaling pathway was performed using Human Apoptosis Antibody Array (R&D Systems, Minneapolis, MN, USA) following the manufacturer’s instructions. Briefly, the transfected T24 cells were collected, washed, and then were lysed by lysis buffer and total proteins were extracted. Protein concentrations were measured by BCA Protein Assay Kit (HyClone-Pierce, Logan, UT, USA). Each array antibody membrane was blocked, then incubated with protein samples (0.5 mg/mL) overnight at 4 °C and continuing incubated with HRP linked Streptavidin conjugate for 1 h. The spots were visualized by enhanced chemiluminescence (ECL) (Amersham, Chicago, IL, USA) and the signal densities were analyzed with ImageJ software (National Institute of Health, Bethesda, MD, USA).

### In vivo tumorigenicity assay

Our animal study was reviewed and approved by Ethics committee of Fudan University. Twenty female 4-week-old BALB/c nude mice were purchased from Shanghai Lingchang Experimental Animals Co., Ltd (Shanghai, China) and randomly divided into two groups (shALPK2 group and shCtrl group). 4 × 10^7^ T24 cells were subcutaneous injected into each mouse for in vivo tumorigenicity. The record of tumor size started 20 days of post-injection and data of weight, *L* and *W* (*L* represent longest dimension and *W* means dimension perpendicular to length) was collected 2–3 times a week (volume of tumor = *π*/6 × *L* × *W*^2^). Tumor burden was assessed weekly by fluorescence imaging and analyzed by IVIS Spectrum Imaging System (Perkin Elmer, Waltham, MA, USA). Mice were sacrificed after 51 days of post-injection. The removed tissues were stored at −80 °C for subsequent experiment.

### Ki67 immunostaining

Tumor tissues removed from mice xenograft models were collected for Ki-67 immunostaining. Tumor tissues were fixed and embedded with formalin and paraffin. Two micrometer slides were cut and immersed in xylene and 100% ethanol for deparaffinization and rehydration, then all slides were blocked with PBS-H_2_O_2_. Ki67 primary antibody was added and incubated with all slides at 4 °C overnight, then all slides were further incubated with the secondary antibody. Finally, all slides were stained by Hematoxylin and Eosin (Baso, Zhuhai, Guangdong, China). Stained slides were examined with a microscopic. The antibodies used were listed in Table S1.

### Statistical analysis

Data are expressed as the mean ± SD and Student’s *t*-test was used to analyze the statistical signifcance. Categorical variables were compared using *χ*^2^ or Fisher’s exact test. Rank sum test analysis was utilized while analysis the expression difference of ALPK2 and Mann–Whitney *U* analysis and Spearman Rank correlation analysis was used, while analyzing the relationship between ALPK2 expression and tumor characteristics in bladder cancer patients. All statistical analysis was performed using SPSS 17.0 (IBM, SPSS, Chicago, IL, USA) and GraphPad Prism 6.01 (Graphpad Software, La Jolla, CA, USA). *P* < 0.05 was considered statistically significant.

## Results

### ALPK2 is upregulated in bladder cancer tissues and expressed in bladder cancer cells

**Fig. 1:**
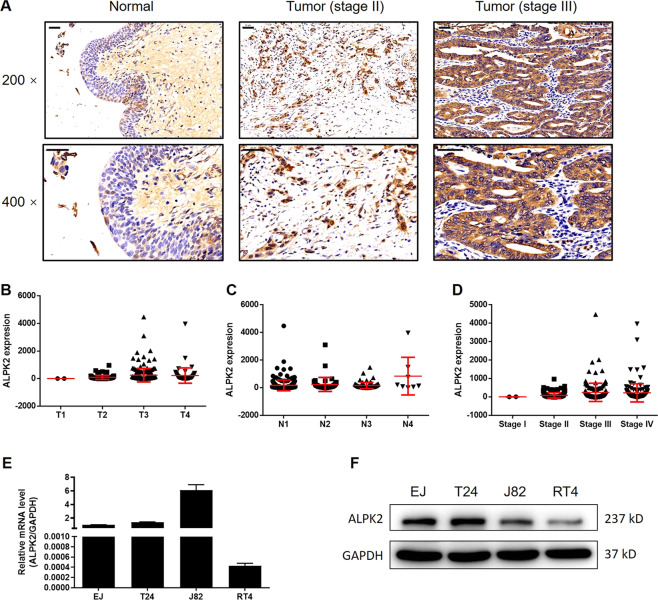
ALPK2 was upregulated in bladder cancer tissues and expressed in bladder cancer cells. **A** The expression level of ALPK2 was detected by IHC analysis in bladder cancer tissues and normal tissues (scale bar = 50 μm). **B**–**D** The expression of ALPK2 in bladder cancer tissues of patients with different T stage, N stage, and pathological stage. **E** The mRNA expression of ALPK2 in EJ, T24, J82, and RT4 cell lines was detected by qPCR. **F** The protein expression of ALPK2 in EJ, T24, J82, and RT4 cell lines was detected by western blot. The representative images were selected from at least three independent experiments. Data were shown as mean ± SD.

In this study, we first investigated the expression levels of ALPK2 in human bladder cancer tissues and then compared these levels with those of normal tissues. IHC analysis showed that the expression levels of ALPK2 in tumor tissues were much higher than those in normal tissues, thus indicating that ALPK2 may be involved in the development and progression of bladder cancer (Fig. 1A and Table 1). Gene expression profiling of The Cancer Genome Atlas (TCGA) database further established a significant correlation between high expression levels of ALPK2 and more advanced T stage, N stage, and pathological stage, in bladder cancer patients (Fig. 1B–D and Tables S3 and S4); these findings were confirmed by our own results, at least to some extent (Fig. 1A). We also used qPCR and western blot to detect the endogenous expression of ALPK2 in various bladder cancer cell lines. As shown in Fig. 1E, F, all of the bladder cancer cell lines tested (EJ, T24, J82, and RT4) showed expression levels of ALPK2, although there were differences in these expression levels across different cell lines. Collectively, these experimental results revealed the potential stimulatory role played by ALPK2 in the development and progression of bladder cancer.

**Table 1:** Expression patterns of ALPK2 in bladder cancer tissues and normal tissues revealed in immunohistochemistry analysis.

ALPK2 expression	Tumor tissue		Normal tissue	
	Cases	Percentage (%)	Cases	Percentage (%)
Low	20	35.7	40	100
High	36	64.3	0	0

*P* < 0.001.

### ALPK2 knockdown inhibited the development of bladder cancer in vitro

**Fig. 2:**
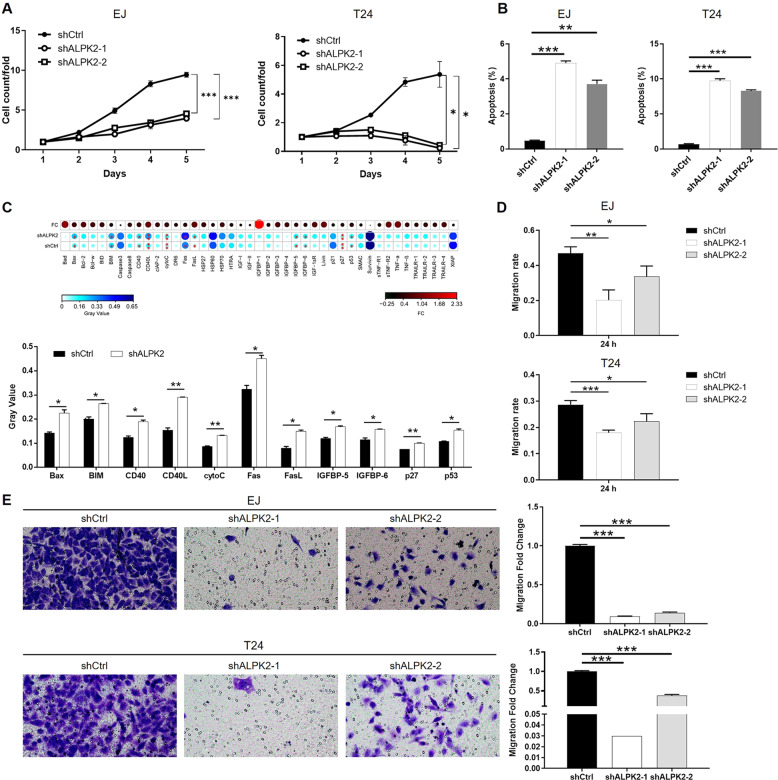
ALPK2 knockdown inhibited bladder cancer development in vitro. **A** Celigo cell counting assay was employed to show the effects of ALPK2 on cell proliferation of EJ and T24 cells. **B** Flow cytometry was performed to detect cell apoptosis of EJ and T24 cells with or without ALPK2 knockdown. **C** Human apoptosis antibody array was utilized to analyze the regulatory ability of ALPK2 on expression of apoptosis-related proteins in T24 cells. **D**, **E** The effects of ALPK2 on cell migration ability of EJ and T24 cells were evaluated by wound-healing assay (**D**) and Transwell assay (**E**). The representative images were selected from at least three independent experiments. Data were shown as mean ± SD. *P < 0.05, **P < 0.01, ***P < 0.001.

In order to explore the role played by ALPK2 in the development and progression of bladder cancer, we prepared lentiviral constructs expressing shRNAs targeting ALPK2 (shALPK2-1 and shALPK2-2) or shCtrl (as a negative control); these constructs were then used to transfect EJ and T24 cells. As shown in Fig. S1, the mRNA levels of ALPK2 were significantly reduced by transfection of shALPK2-1 and shALPK2-2 in EJ and T24 cells (*P* < 0.01), respectively. The depletion of ALPK2 was also verified by detecting ALPK2 protein in EJ and T24 cells by western blotting (Fig. S1). Next, we demonstrated that bladder cancer cells exhibiting downregulated expression levels of ALPK2 (shALPK2) also showed significantly slower proliferation rates than the shCtrl group (*P* < 0.05, Figs. 2A and S2A). The proportion of apoptotic EJ and T24 cells (in which ALPK2 had been knocked down) was significantly higher than that in cells transfected with shCtrl (*P* < 0.01, Figs. 2B and S2B). Next, we used a Human Apoptosis Antibody Array on T24 cells with or without ALPK2 to identify the regulatory effects of ALPK2 knockdown on apoptosis-related proteins, This demonstrated the upregulation of Bax, BIM, CD40, CD40L, cytoC, Fas, FasL, IGFBP-5, IGFBP-6, p27, and p53 (*P* < 0.05, Figs. 2C and S2C). Collectively, ALPK2 regulated both cell proliferation and apoptosis. Wound-healing assays further revealed a significant suppression of the migration ability of EJ and T24 cells in the shALPK2 groups (at 24 h, *P* < 0.05, Figs. 2D and S3); these results were further confirmed by Transwell assays (*P* < 0.001, Fig. 2E). In summary, these in vitro studies clearly showed that ALPK2 plays an important role in the development and progression of bladder cancer.

### ALPK2 knockdown inhibited the tumor growth of bladder cancer in vivo

**Fig. 3:**
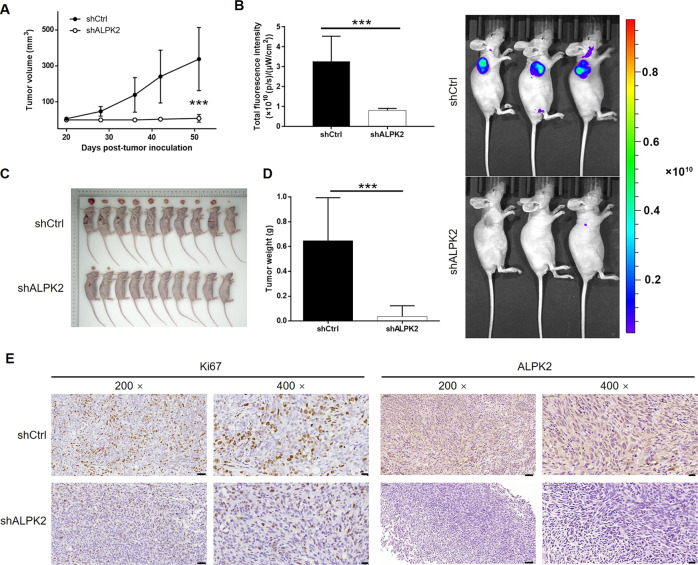
ALPK2 knockdown inhibited bladder cancer development in vivo. **A** Twenty days of post-injection of T24 cells with or without ALPK2 knockdown, the volume of tumors formed in mice was measured and calculated at indicated time intervals. **B** In vivo imaging was performed to evaluate the tumor burden in mice of shALPK2 and shCtrl groups at day 22 of post-tumor-inoculation. The fluorescence intensity was scanned and used as a representation of tumor burden in mice of shALPK2 and shCtrl groups. **C**, **D** Mice were sacrificed at day 22 of post-injection, and the tumors were removed for collecting photos (**C**) and weighing (**D**). **E** The expression of Ki67 and ALPK2 in tumors removed from mice of both groups was detected by IHC analysis (scale bar = 50 μm in 200 magnification, scale bar = 20 μm in 400 magnification). Data were shown as mean ± SD. ***P < 0.001.

In order to further investigate the influence of ALPK2 knockdown on tumor growth in vivo, we constructed mice xenograft models via the subcutaneous injection of T24 cells transfected with either shALPK2 (shALPK2-1) or shCtrl. We then measured tumor volume, beginning on day 7 of post-inoculation; analysis showed that tumors in the shALPK2 group had a significantly slower growth rate. The final volume (day 22 of post-inoculation) of tumors formed in the shALPK2 group was significantly smaller than those in the shCtrl group (*P* < 0.001, Fig. 3A). We also assessed the tumor burden of mice in the shALPK2 and shCtrl groups by in vivo imaging, facilitated by the fluorescent labels on lentivirus vector. Mice in the shALPK2 group, showed far stronger levels of fluorescence ALPK2 than mice from the shCtrl group, thus indicating that the knockdown of ALPK2 suppressed the growth of tumors in vivo (*P* < 0.001, Fig. 3B). The inhibition of bladder cancer development was also verified by directly observing the tumors removed from sacrificed animals, and the significantly lower weight of tumors removed from mice in the shALPK2 group (*P* < 0.001, Fig. 3C, D). In addition, ALPK2-induced inhibition of tumor growth was also supported by the lower Ki67 in tumors formed by T24 cells transfected with shALPK2, as well as the downregulation of ALPK2 in shALPK2 xenografts (Fig. 3E). Collectively, these results strongly confirmed the role of ALPK2 in the development and progression of bladder cancer, both in vitro and in vivo.

### The potential of DEPDC1A as a downstream target of ALPK2 in the regulation of bladder cancer

**Fig. 4:**
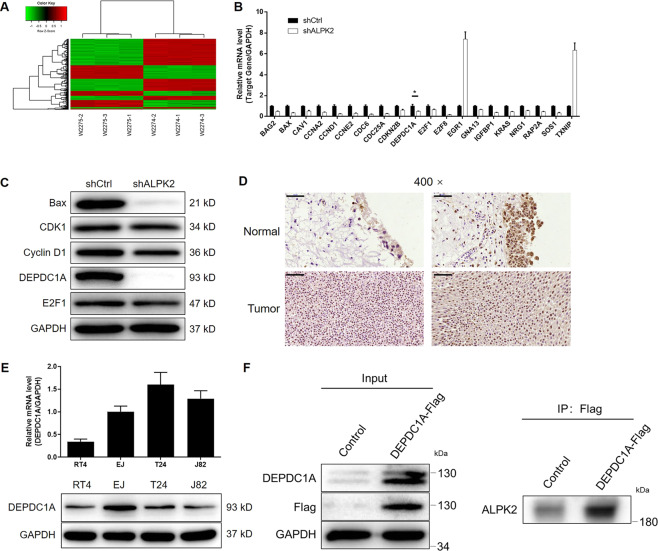
The exploration and verification of downstream underlying ALPK2 induced regulation of bladder cancer. **A** A PrimeView Human Gene Expression Array was performed to identify the differentially expressed genes (DEGs) between shALPK2 and shCtrl groups of T24 cells. qPCR (**B**) and western blotting (**C**) were used to detect the expression of several selected DEGs in T24 cells with or without ALPK2. **D** The expression of DEPDC1A in bladder cancer tissues and normal tissues was evaluated by IHC analysis (scale bar = 50 μm in 200 magnification, scale bar = 20 μm in 400 magnification). **E** The mRNA and protein expression of ALPK2 in EJ, T24, J82, and RT4 cell lines was detected by qPCR and western blot, respectively. **F** The interaction between ALPK2 protein and DEPDC1A protein was confirmed by co-immunoprecipitation assay. The representative images were selected from at least three independent experiments. Data were shown as mean ± SD. *P < 0.05.

Given the evident role of ALPK2 in the development of bladder cancer, we next explored the downstream mechanisms that might be responsible for its regulatory effects on bladder cancer. We performed a PrimeView Human Gene Expression Array to identify differentially expressed genes (DEGs) between the shALPK2 and shCtrl groups of T24 cells. In total, 684 DEGs were identified based on a specific threshold (absolute fold change > 2 and FDR < 0.05), including 257 DEGs that were upregulated and 427 DEGs that were downregulated in the shALPK2 group (Figs. 4A, S4A, and S4B). Enrichment analysis, based on all of these DEGs, further revealed that the most enriched canonical signaling pathways were those referred to as “molecular mechanisms of cancer” (Fig. S4C), while IPA analysis showed that the most enriched IPA diseases and functions were “cancer”, “organismal injury and abnormalities” (Fig. S4D). Twenty DEGs were selected for qPCR detection in EJ cells (Fig. 4B), and five DEGs were subjected to western blotting for further verification (Fig. 4C). We found that the mRNA and protein levels of DEPDC1A were downregulated DEPDC1Ain bladder cells in which ALPK2 had been knocked down, thus suggesting that DEPDC1A might represent a potential target for ALPK2. In order to confirm this hypothesis. Then, we conducted IHC analysis (Fig. 4D) to detect the expression levels of DEPDC1A in bladder cancer tissues, and compared these levels with those in normal tissues; we found that DEPDC1A was upregulated in bladder cancer. Moreover, we found that, similar with ALPK2, DEPDC1A is expressed in EJ, T24, J82, and RT4 cells (Fig. 4E). More importantly, in order to preliminarily investigate the regulatory mechanism between ALPK2 and DEPDC1A, a co-immunoprecipitation assay was carried out and showed that ALPK2 and DEPDC1A possessed protein-protein interaction (Fig. 4F). On the other hand, the detection of DEPDC1A expression in xenografts also showed the downregulated DEPDC1A expression in shALPK2 xenografts (Fig. S4E).

### Knockdown of DEPDC1A blocked the development of bladder cancer in vitro

**Fig. 5:**
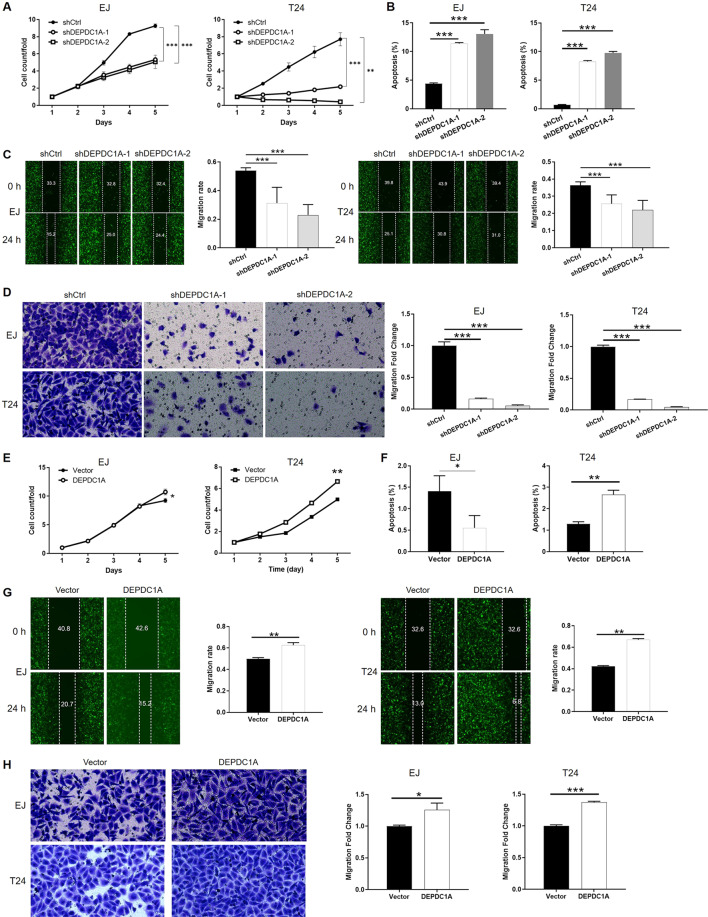
DEPDC1A regulated bladder cancer development in vitro. **A**, **E** Celigo cell counting assay was employed to show the effects of DEPDC1A on cell proliferation of EJ and T24 cells. **B**, **F** Flow cytometry was performed to detect cell apoptosis of EJ and T24 cells with or without DEPDC1A knockdown (**B**)/overexpression (**F**). **C**, **D**, **G**, **H** The effects of DEPDC1A on cell migration ability of EJ and T24 cells were evaluated by wound-healing assay (**C**, **G**) and Transwell assay (**D**, **H**). The representative images were selected from at least three independent experiments. Data were shown as mean ± SD. *P < 0.05, **P < 0.01, ***P < 0.001.

In order to investigate the role of DEPDC1A in the development of bladder cancer, we carried out a range of cell phenotypes detections in EJ and T24 cells in which DEPDC1A was knocked down. Knockdown efficiency of DEPDC1A was evaluated by a combination of qPCR and western blotting (*P* < 0.01, Fig. S5). Celigo cell counting assay indicated that the cell growth of EJ and T24 cells transfected with shDEPDC1A had almost stopped, while the cells transfected with shCtrl grew normally (*P* < 0.01, Figs. 5A and S6A). The effects of DEPDC1A knockdown on cell apoptosis were also similar to those produced by the knockdown of ALPK2; there was a significantly higher proportion of apoptotic cells in the shDEPDC1A groups (*P* < 0.001, Figs. 5B and S6B). Finally, wound-healing assays and Transwell assays demonstrated that bladder cancer cells in which DEPDC1A had been knocked down had significantly weaker motility (*P* < 0.001, Fig. 5C, D). In summary, DEPDC1A exhibited similar regulatory effects on the development of bladder cancer as ALPK2. However, the precise association between these two factors has yet to be elucidated.

### DEPDC1A overexpression partially reversed the tumor inhibition induced by ALPK2 knockdown

**Fig. 6:**
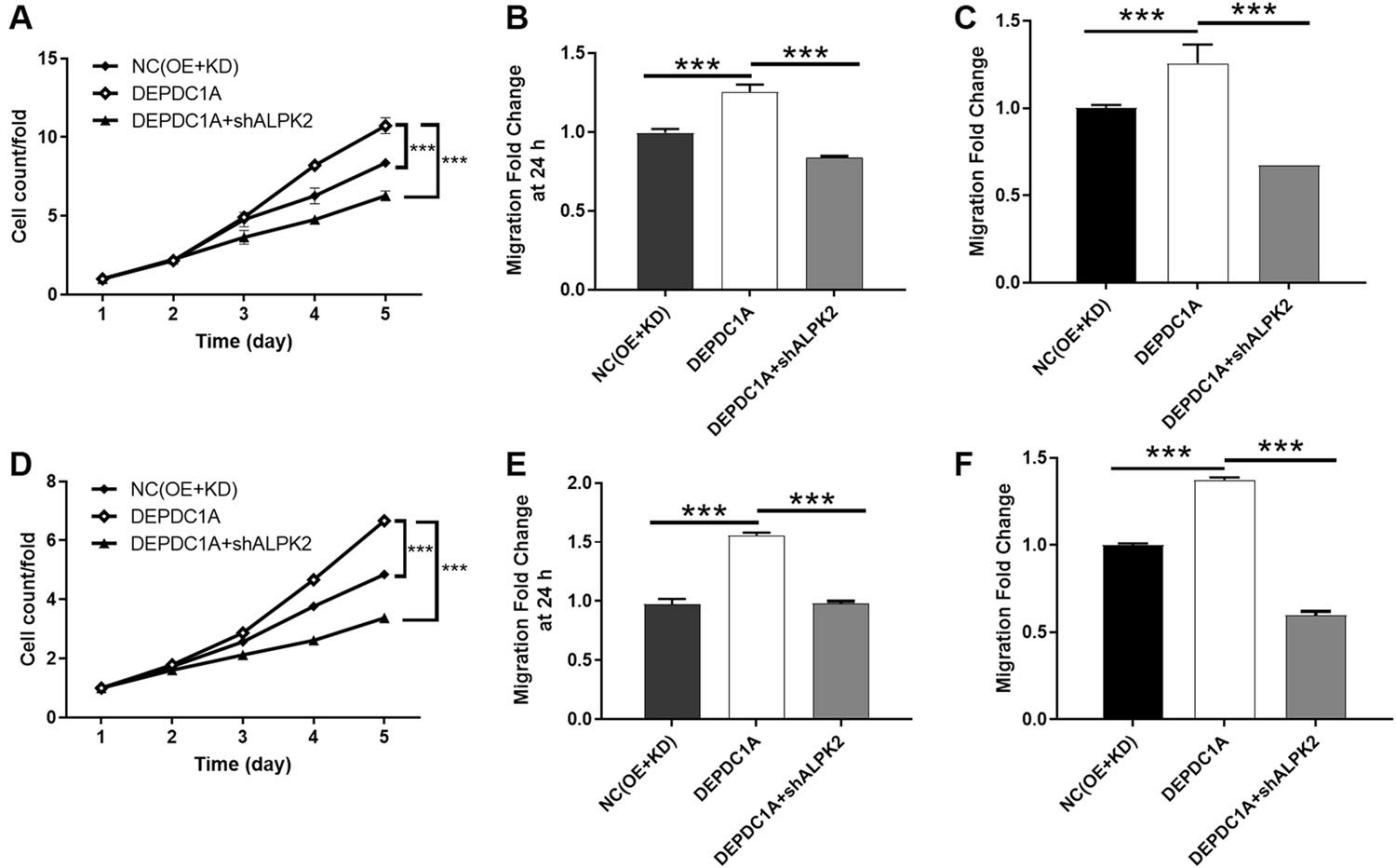
Overexpression of DEPDC1A attenuated the effects on bladder cancer cells by ALPK2 knockdown. EJ and T24 cell models with mere DEPDC1A overexpression or simultaneous DEPDC1A overexpression and ALPK2 knockdown were subjected to the detection of cell proliferation by Celigo cell counting assay (**A**, **D**), cell migration by wound-healing assay **(B**, **E**) and cell migration by Transwell assay (**C**, **F**). Data were shown as mean ± SD. ***P < 0.001.

Next, we constructed EJ and T24 cells that overexpressed DEPDC1A, and cells in which ALPK2 was knocked down while DEPDC1A was overexpressed, and used these groups of cells to investigate the synergistic effects of DEPDC1A and ALPK2 on bladder cancer. First, we demonstrated the successful overexpression of DEPDC1A in EJ and T24 cells by qPCR and western blot (*P* < 0.05, Fig. S7); these cells also proliferated significantly faster (*P* < 0.05, Figs. 5E and S8A). Interestingly, we expected to witness the inhibition of apoptosis but failed in T24 cells; this may be attributed to the low apoptosis rate in the shCtrl group (Figs. 5F and S8B). Furthermore, the overexpression of DEPDC1A promoted cell motility of EJ and T24 cells, as determined by wound-healing and Transwell assays (*P* < 0.05, Fig. 5G, H). More importantly, EJ and T24 cell models with simultaneous DEPDC1A overexpression and ALPK2 knockdown (DEPDC1A + shALPK2) were constructed and verified (*P* < 0.01, Fig. S9). The comparison of the results obtained from DEPDC1A group (DEPDC1A overexpression) and the DEPDC1A + shALPK2 group (DEPDC1A overexpression + ALPK2 knockdown) group demonstrated that all of the effects induced by ALPK2 knockdown on cell proliferation (*P* < 0.001, Fig. 6A, D), and cell migration (*P* < 0.001, wound healing assay in Fig. 6B, D, Transwell assay in Fig. 6C, F), could be alleviated, or even reversed, by the overexpression of DEPDC1A. Collectively, these results clearly demonstrated the promotion effects of ALPK2 on bladder cancer, and that DEPDC1A may also be involved.

## Discussion

As one of the six alpha kinases, ALPK2 has been well known as a member of an atypical alpha protein kinase family. Till now, studies concerning the biological functions of ALPK2 are still limited. Through loss-of-function siRNA assay, Murry’s group proposed that ALPK2 could act as an inhibitor of Wnt/β-catenin signaling pathway, thus participating the cardiac development [22]. They further demonstrated that ALPK2 may play a key role during the transition of human embryonic stem cells (hESCs) from mesoderm to cardiac progenitors, and knockdown of ALPK2 could inhibit cardiac function and cardiomyocyte differentiation. Evidence that ALPK2 was involved in cardiac development through negatively regulating Wnt/β-catenin signaling pathway was also showed in this work [22]. Besides, in human ovarian cancer, the mutation of *ALPK2* gene was found by through sequence-based analysis Fearon et al., indicating its potential function in human cancers [18]. Furthermore, Shirasawa et al. not only discovered the differential expression of ALPK2 between HCT116 and HKe3 cells in a three-dimensional (3D) colonic-crypt model, but also revealed its involvement in luminal apoptosis, DNA repair-related gene expression and possibly the colonic crypt to adenoma transition [23]. However, the role in the development and progression of bladder cancer played by ALPK2 is rarely reported and still largely unknown till now.

In this study, it was found that the expression of ALPK2 in bladder cancer tissues was generally higher than that in normal tissues, indicating its potential role as a tumor promotor in bladder cancer. Subsequent in vitro experiments validated the role of ALPK2 in bladder cancer via uncovering the inhibition of cell proliferation and cell motility, and the promotion of cell apoptosis induced by ALPK2 knockdown. Furthermore, the regulation of cell apoptosis by ALPK2 knockdown was further rationalized by its capability of upregulating apoptosis-related proteins including Bax, BIM, CD40, CD40L, cytoC, Fas, FasL, IGFBP-5, IGFBP-6, p27, and p53. The observation and measurement of tumor-bearing mice models further make clear the promotor role of ALPK2 in the development and progression of bladder cancer, which was represented by slower growth rate and smaller final tumors formed by bladder cancer cells with ALPK2 depletion, merely with an unclear mechanism.

DEP domain is a protein motif consisting of about 100 amino acid residues [24]. This conserved sequence was first defined and named based on three proteins: Disevelled, EGL-10 and Peckstrin 3 [24, 25]. Till now, it has been proved that DEP domain has a variety of biological functions, such as signal transduction, cell polarity determination, cell anchoring, and so on [26]. More importantly, recent studies showed that DEP domain containing 1A (DEPDC1A), alias DEPDC1, was tightly correlated with the development and prognosis of several types of human cancers [27–30]. It has been illustrated that the complex formed by DEPDC1A and zinc finger protein 224 (ZNF224) inhibited the expression of zinc finger protein A20, weakening the inhibitory effect of A20 on degradation of inhibitor of NF-κB (I-κB), thus promoting the translocation of NF-κB into the nucleus to activate the expression of related genes, and ultimately playing an anti-apoptotic physiological role in bladder cancer [31]. Actually, similar effects and mechanism were also revealed in hepatocellular carcinoma and lung adenocarcinoma [32, 33]. Moreover, the expression of DEPDC1A was also found to be of prognostic significance in patients with hepatocellular carcinoma or breast cancer [29, 34–36]. Besides, DEPDC1A was also reported to exert essential functions in nasopharyngeal carcinoma [37], prostate cancer [30, 38], glioma [39], and endometrial endometrioid carcinoma [40]. In this study, combining the bioinformatics of gene expression profiling of bladder cancer cells with or without ALPK2 knockdown and its known role in bladder cancer, DEPDC1A was identified as a potential target of the ALPK2 induced regulation of bladder cancer. Our studies further confirmed the significantly upregulated expression of DEPDC1A in bladder cancer tissues compared with normal ones. Although the oncogenic role of DEPDC1A in bladder cancer has been elucidated by previous work, the capacity of DEPDC1A knockdown to suppress bladder cancer cell growth, cell motility, and to promote cell apoptosis was manifested in this study. In contrast, the promotion effects of DEPDC1A overexpression on cell proliferation and cell migration were also proved. More importantly, our results provided valid evidence that DEPDC1A overexpression could alleviate or even reverse the influence of ALPK2 knockdown on bladder cancer cell proliferation and cell migration ability, indicating the involvement of DEPDC1A in the regulation of bladder cancer induced by ALPK2.

In conclusion, our study revealed that ALPK2 possessed the ability of promoting development and progression of bladder cancer through the regulation of oncoprotein DEPDC1A. Therefore, ALPK2 may act as a tumor promotor in bladder cancer, and could be used as a potential therapeutic target in the treatment of bladder cancer.

## Supplementary information

supplementary figure legends

Table S1

Table S2

Table S3

Table S4

Figure S1

Figure S2

Figure S3

Figure S4

Figure S5

Figure S6

Figure S7

Figure S8

Figure S9

## Data Availability

All data generated or analyzed during this study are included in this published article and its supplementary information files.
