# Supplementary material for: ALPK2 acts as tumor promotor in development of bladder cancer through targeting DEPDC1A
Source: Cell Death Dis. 2021 Jul 1;12(7):661. doi: 10.1038/s41419-021-03947-7 (PMC8249393; doi:10.1038/s41419-021-03947-7)
Supplement: Supplementary file 1 — supplementary figure legends [file 41419_2021_3947_MOESM1_ESM.docx]

**Figure S1.** EJ and T24 cell models with or without ALPK2 knockdown were constructed by transfecting shALPK2 or shCtrl. The knockdown efficiency of ALPK2 in EJ and T24 cells was assessed by qPCR and western blotting, respectively. Data were shown as mean ± SD. ***P* < 0.01, ****P* < 0.001

**Figure S2.** (A) Representative images of cell proliferation detected by celigo cell counting assay. (B) Representative images of apoptosis determined by flow cytometry. (C) Human Apoptosis Antibody Array was performed to detect and compare the expression of apoptosis-related proteins in T24 cells with or without ALPK2 knockdown.

**Figure S3.** Representative images of cell migration detected by wound-healing assay.

**Figure S4.** (A) The scatter plot of gene expression profiling in T24 cells with or without ALPK2 knockdown. Red dots represented significantly upregulated DEGs. Green dots represented significantly downregulated DEGs. (B) The volcano plot of gene expression profiling in T24 cells with or without ALPK2 knockdown. Red dots represented the DEGs. (C) The enrichment of the DEGs in canonical signaling pathways was analyzed by IPA. (D) The enrichment of the DEGs in IPA disease and function was analyzed by IPA. (E) The expression of DEPDC1A in tumors removed from mice of both groups was detected by IHC analysis (scale bar = 50 μm in 200 magnification, scale bar = 20 μm in 400 magnification). Data were shown as mean ± SD. **P* < 0.05, ***P* < 0.01, ****P* < 0.001

**Figure S5.** EJ and T24 cell models with or without DEPDC1A knockdown were constructed by transfecting shDEPDC1A or shCtrl. The knockdown efficiency of DEPDC1A in EJ and T24 cells was assessed by qPCR and western blotting, respectively. Data were shown as mean ± SD. ***P* < 0.01

**Figure S6.** (A) Representative images of cell proliferation detected by celigo cell counting assay. (B) Representative images of apoptosis determined by flow cytometry.

**Figure S7.** EJ and T24 cell models with or without DEPDC1A overexpression were constructed by transfecting DEPDC1A overexpression plasmids or vector. The overexpression of DEPDC1A in EJ and T24 cells was verified by qPCR and western blotting, respectively. Data were shown as mean ± SD. **P* < 0.05

**Figure S8.** (A) Representative images of cell proliferation detected by celigo cell counting assay. (B) Representative images of apoptosis determined by flow cytometry.

**Figure S9.** The expression of ALPK2 and DEPDC1A in EJ and T24 cells with simultaneous DEPDC1A overexpression and ALPK2 knockdown was detected by qPCR and western blot, respectively. (A, B) EJ cells; (C, D) T24 cells. Data were shown as mean ± SD. ***P* < 0.01, ****P* < 0.001
