# Supplementary material for: ALPK2 acts as tumor promotor in development of bladder cancer through targeting DEPDC1A
Source: Cell Death Dis. 2021 Jul 1;12(7):661. doi: 10.1038/s41419-021-03947-7 (PMC8249393; doi:10.1038/s41419-021-03947-7)
Supplement: Supplementary file 2 — Table S1 [file 41419_2021_3947_MOESM2_ESM.docx]

Table S1 Antibodies used in western blotting and IHC

| Primary antibodies | Dilution in WB | Source species | Company | Catalog No. |
| --- | --- | --- | --- | --- |
| ALPK2 | 1:1000 | Rabbit | abcam | ab111909 |
| GAPDH | 1:3000 | Rabbit | Bioworld | AP0063 |
| Bax | 1:2000 | Rabbit | abcam | ab32503 |
| CDK1 | 1:2000 | Rabbit | abcam | ab133327 |
| Cyclin D1 | 1:2000 | Rabbit | CST | 2978 |
| E2F1 | 1:1000 | Rabbit | abcam | ab179445 |
| DEPDC1A | 1:500 | Rabbit | abcam | ab197246 |
|  |  |  |  |  |
| Primary antibodies | Dilution in IHC | Source species | Company | Catalog No. |
| ALPK2 | 1:200 | Rabbit | abcam | ab111909 |
| Ki67 | 1:200 | Rabbit | abcam | ab16667 |
| DEPDC1A | 1:100 | Rabbit | abcam | ab197246 |
|  |  |  |  |  |
|  |  |  |  |  |
| Secondary antibody | Dilution |  | Company | Catalog No. |
| HRP Goat Anti-Rabbit IgG (WB) | 1:3000 |  | Beyotime | A0208 |
| HRP Goat Anti-Rabbit IgG (IHC) | 1:200 |  | Abcam | Ab111909 |
