# Supplementary material for: ALPK2 acts as tumor promotor in development of bladder cancer through targeting DEPDC1A
Source: Cell Death Dis. 2021 Jul 1;12(7):661. doi: 10.1038/s41419-021-03947-7 (PMC8249393; doi:10.1038/s41419-021-03947-7)
Supplement: Supplementary file 3 — Table S2 [file 41419_2021_3947_MOESM3_ESM.docx]

Table S2 Primers used in qPCR

| Gene | Forward primer sequence (5’-3’) | Reverse primer sequence (5’-3’) |
| --- | --- | --- |
| BAG2 | CAGCAGCAAGAATCCCTAAAGC | CATGTGGCACCTCAGATGAAC |
| BAX | CCCTTTTGCTTCAGGGTTTC | TCGCTCAGCTTCTTGGTGGA |
| CAV1 | TCTGGGGCATTTACTTCGC | GATGGAATAGACACGGCTGATG |
| CCNA2 | AGCCTGCGTTCACCATTCA | GGGCATCTTCACGCTCTATTTT |
| CCND1 | AAAGAATTTGCACCCCGCTG | GACAGACAAAGCGTCCCTCA |
| CCNE2 | TTGGCTATGCTGGAGGAAGTAA | TTCAGTGCTCTTCGGTGGTG |
| CDC6 | CCAGGCACAGGCTACAATCAGT | AGAACAAGGAGGTAAATGGGGAG |
| CDC25A | TGGAAGTACAAAGAGGAGGAAGAG | GCCAGGGATAAAGACTGATGAAG |
| CDKN2B | GCGAGGAGAACAAGGGCA | GCACCTTCTCCACTAGTCCC |
| DEPDC1A | AATGGGTACGAGGTCACTGATGA | TGAGTAAGTTGGCAAAGGAGCA |
| E2F1 | CACTTTCGGCCCTTTTGCTC | GTGCTCTCACCGTCCTACAC |
| E2F8 | TGACGAAGTGGCAGAGGAAC | CATCATAATCTGCTCGGCGTA |
| EGR1 | CACCTGACCGCAGAGTCTTTT | TGGTTTGGCTGGGGTAACTG |
| GNA13 | AGATGATGTCGTTTGATACCCG | CGCTGTCTGCCCATAATGCT |
| IGFBP1 | CCAAGGCACAGGAGACATCA | ATTCCAAGGGTAGACGCACC |
| KRAS | GACTGGGGAGGGCTTTCTTT | CTAAGTCCTGAGCCTGTTTTGTG |
| NRG1 | CCATCACCCTCAGCAGTTCA | GGTCGTTTCATACTCCTCATCCT |
| RAP2A | CAGGGCTTCATCCTCGTCTACA | TGACTGGCACTTTCTCATACCG |
| SOS1 | GCCAGCCTCATTGTCCCTAA | ACTGAAGGGGGTCCAATGTG |
| TXNIP | TGTTCCCGAATTGTGGTCC | TGCGCATGTCCCTGAGATAA |
| GAPDH | TGACTTCAACAGCGACACCCA | CACCCTGTTGCTGTAGCCAAA |
| ALPK2 | TCCGAAGGACCAGGGACTCTAT | CGGTGAACCCCTTCTCCAAA |
