# Supplementary material for: ALPK2 acts as tumor promotor in development of bladder cancer through targeting DEPDC1A
Source: Cell Death Dis. 2021 Jul 1;12(7):661. doi: 10.1038/s41419-021-03947-7 (PMC8249393; doi:10.1038/s41419-021-03947-7)
Supplement: Supplementary file 4 — Table S3 [file 41419_2021_3947_MOESM4_ESM.docx]

Table S3 Relationship between ALPK2 expression and tumor characteristics in patients with bladder cancer

| Features | No. of patients | ALPK2 expression | | *P* value |
| --- | --- | --- | --- | --- |
|  |  | low | high |  |
| All patients | 404 | 201 | 203 |  |
| T stage |  |  |  | <0.001 |
| T1 | 2 | 2 | 0 |  |
| T2 | 145 | 96 | 49 |  |
| T3 | 199 | 78 | 121 |  |
| T4 | 58 | 25 | 33 |  |
| N stage |  |  |  | <0.001 |
| N0 | 233 | 123 | 110 |  |
| N1 | 46 | 19 | 27 |  |
| N2 | 75 | 29 | 46 |  |
| N3 | 8 | 1 | 7 |  |
| Pathological stage |  |  |  | 0.00619 |
| Stage I | 2 | 2 | 0 |  |
| Stage II | 126 | 87 | 39 |  |
| Stage III | 140 | 56 | 84 |  |
| Stage IV | 134 | 55 | 79 |  |
|  |  |  |  |  |
|  |  |  |  |  |
|  |  |  |  |  |
|  |  |  |  |  |
|  |  |  |  |  |
|  |  |  |  |  |
|  |  |  |  |  |
|  |  |  |  |  |
