# Supplementary material for: ALPK2 acts as tumor promotor in development of bladder cancer through targeting DEPDC1A
Source: Cell Death Dis. 2021 Jul 1;12(7):661. doi: 10.1038/s41419-021-03947-7 (PMC8249393; doi:10.1038/s41419-021-03947-7)
Supplement: Supplementary file 5 — Table S4 [file 41419_2021_3947_MOESM5_ESM.docx]

Table S4 Relationship between ALPK2 expression and tumor characteristics in patients with bladder cancer analyzed by Pear correlation analysis

| Tumor characteristics | index |  |
| --- | --- | --- |
| T stage | Pearson correlation | 0.2286 |
|  | Significance (two tailed) | <0.001 |
|  | n | 404 |
| N stage | Pearson correlation | 0.1496 |
|  | Significance (two tailed) | 0.0043 |
|  | n | 404 |
| Pathological stage | Pearson correlation | 0.2286 |
|  | Significance (two tailed) | <0.001 |
|  | n | 404 |
