# Supplementary figures and images for: ALPK2 acts as tumor promotor in development of bladder cancer through targeting DEPDC1A
Source: Cell Death Dis. 2021 Jul 1;12(7):661. doi: 10.1038/s41419-021-03947-7 (PMC8249393; doi:10.1038/s41419-021-03947-7)

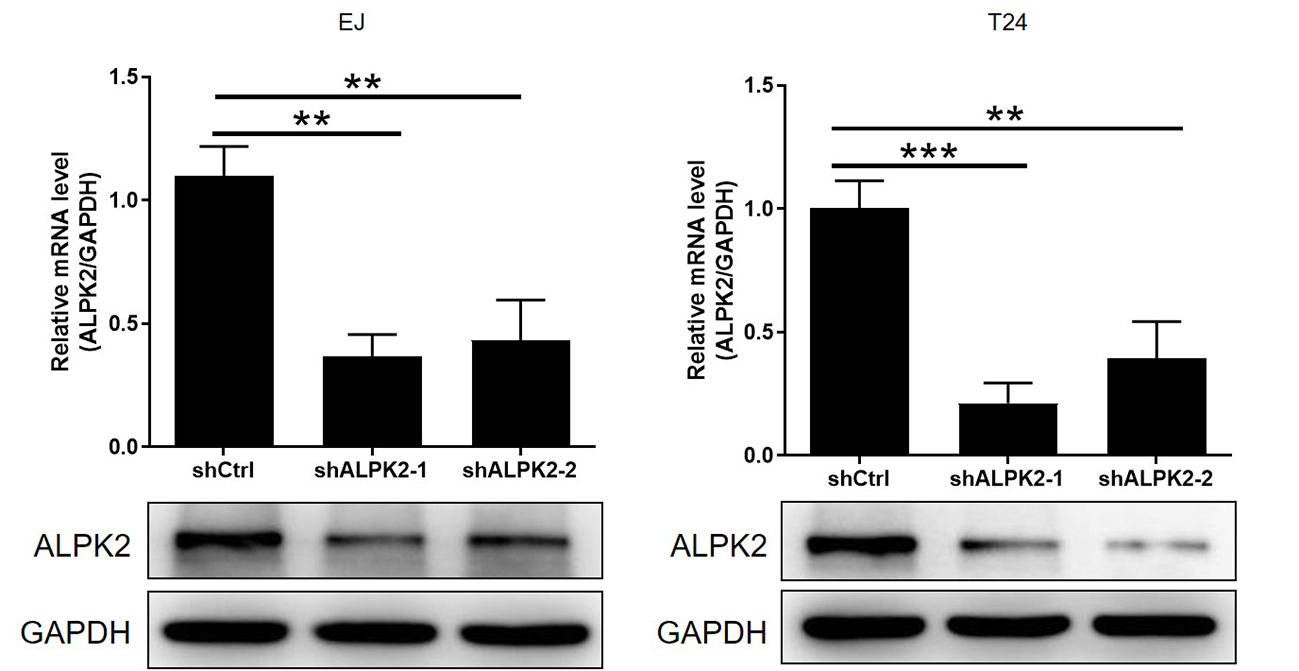

Supplement: Supplementary file 6 — Figure S1 [file 41419_2021_3947_MOESM6_ESM.tif]

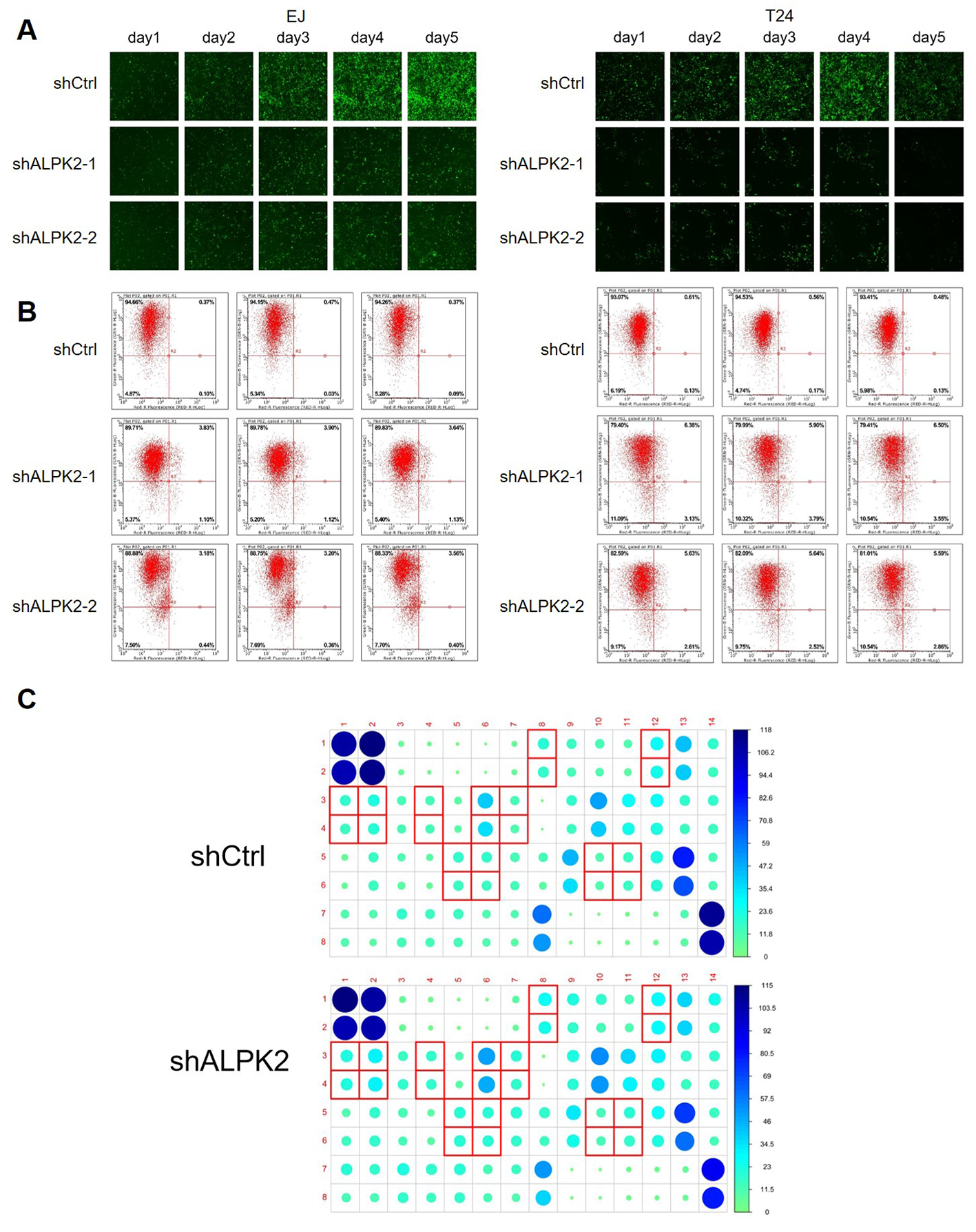

Supplement: Supplementary file 7 — Figure S2 [file 41419_2021_3947_MOESM7_ESM.tif]

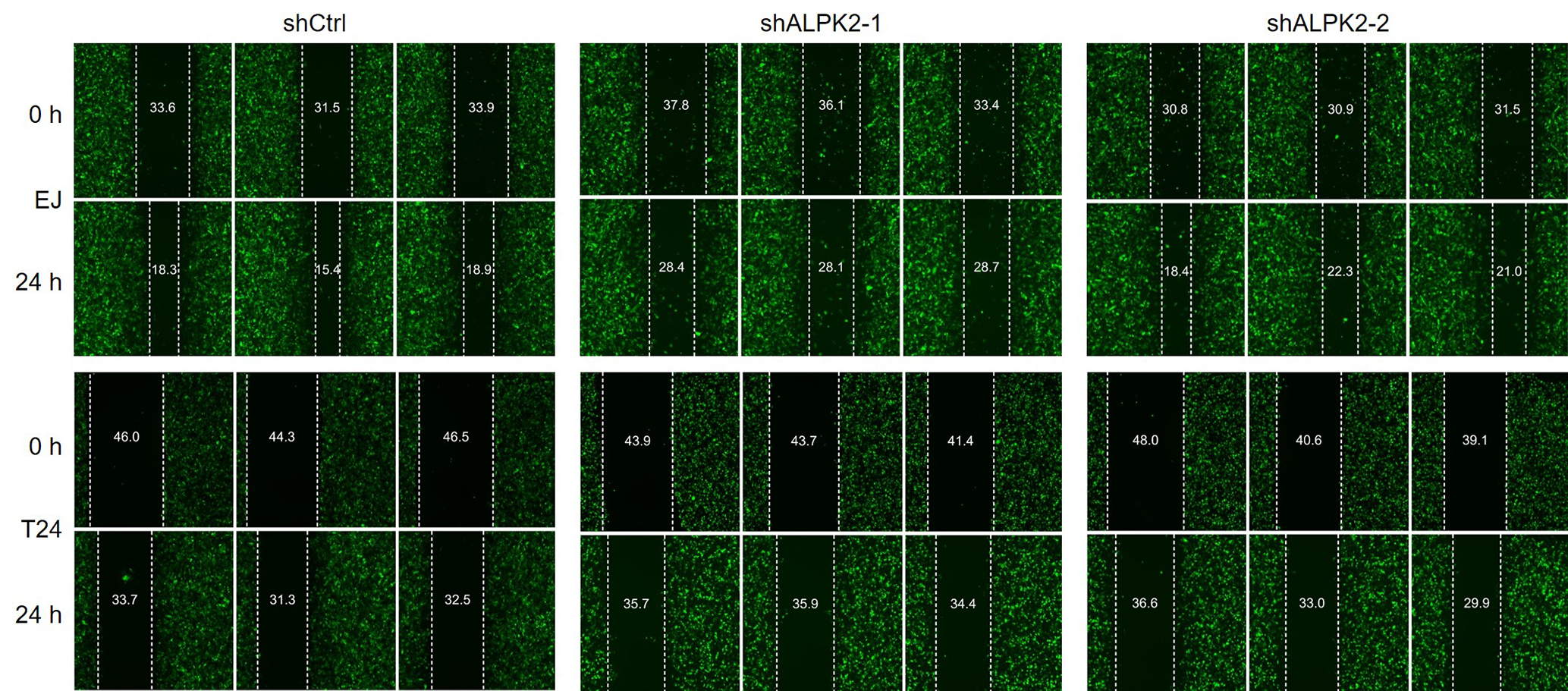

Supplement: Supplementary file 8 — Figure S3 [file 41419_2021_3947_MOESM8_ESM.tif]

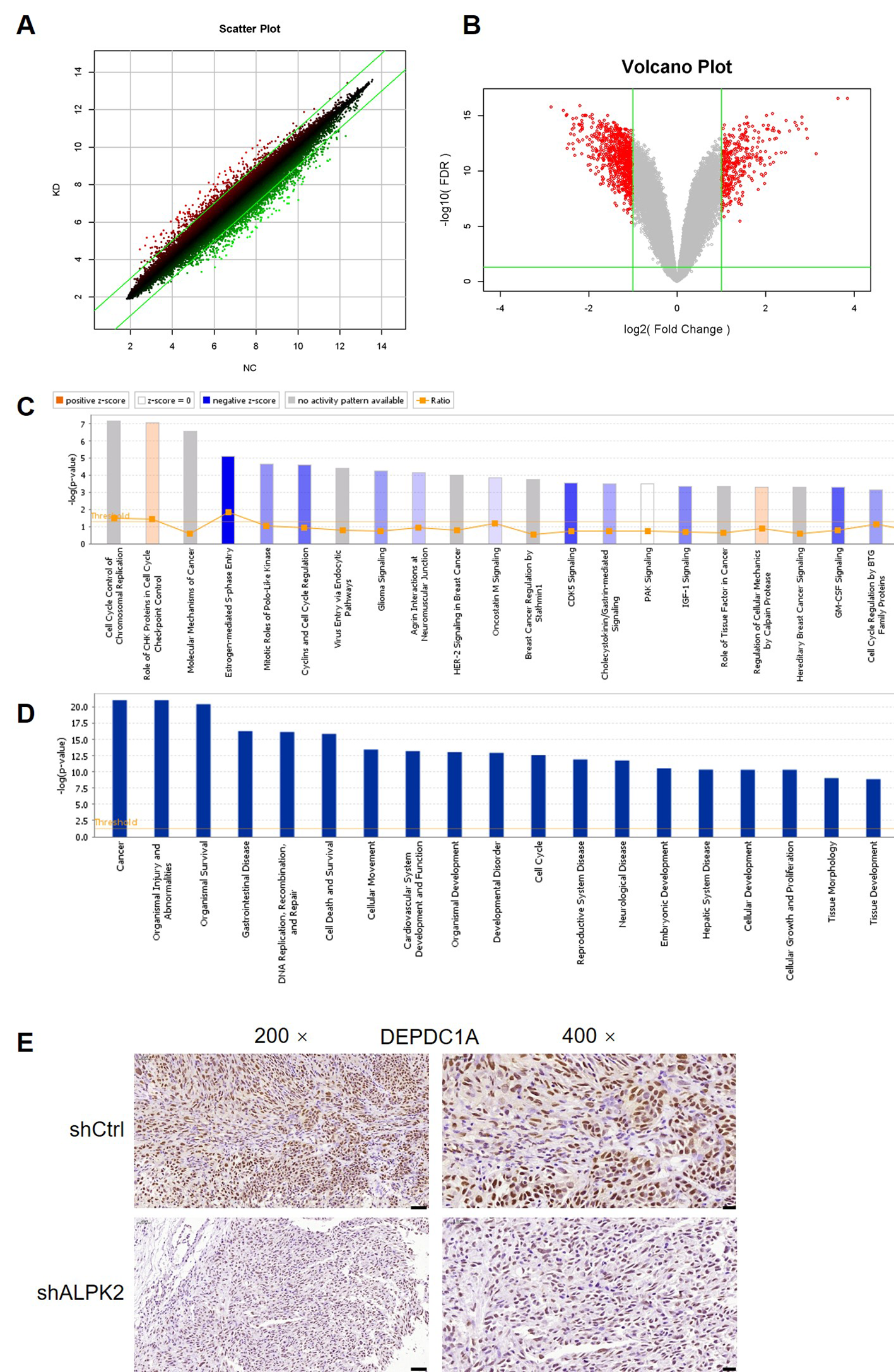

Supplement: Supplementary file 9 — Figure S4 [file 41419_2021_3947_MOESM9_ESM.tif]

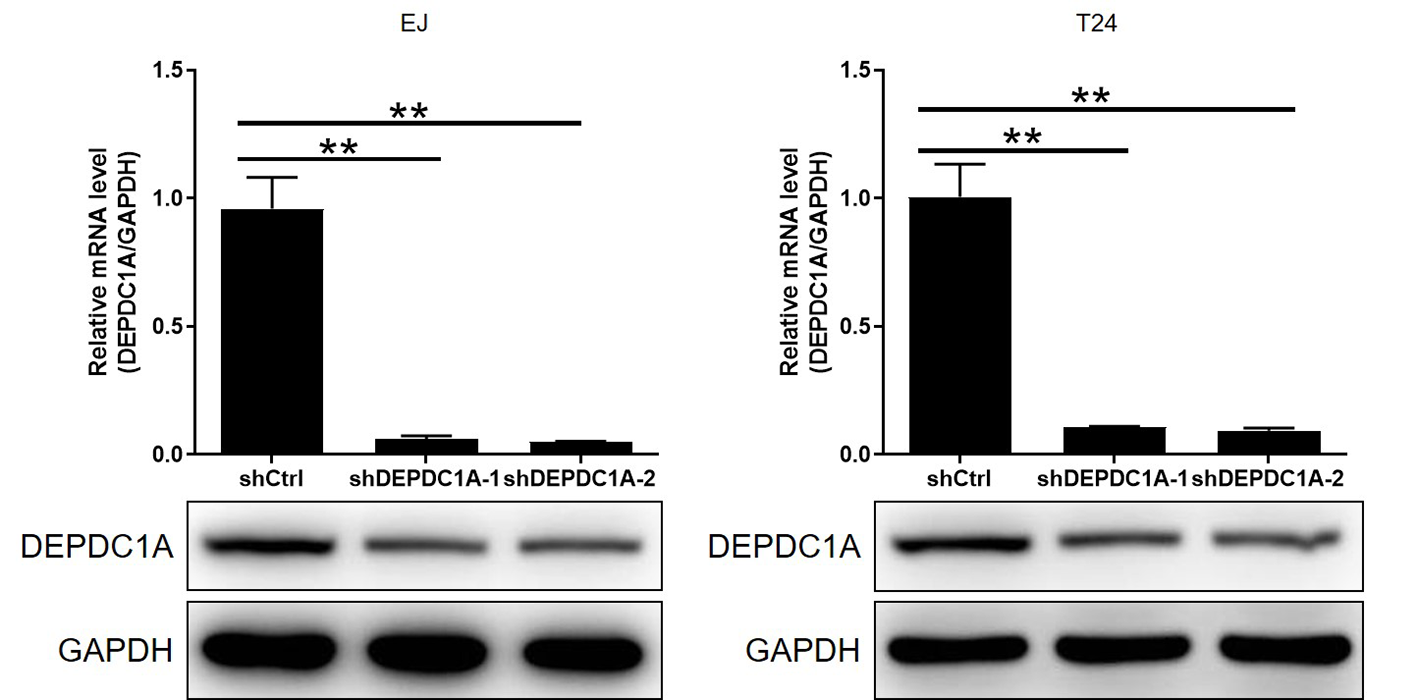

Supplement: Supplementary file 10 — Figure S5 [file 41419_2021_3947_MOESM10_ESM.tif]

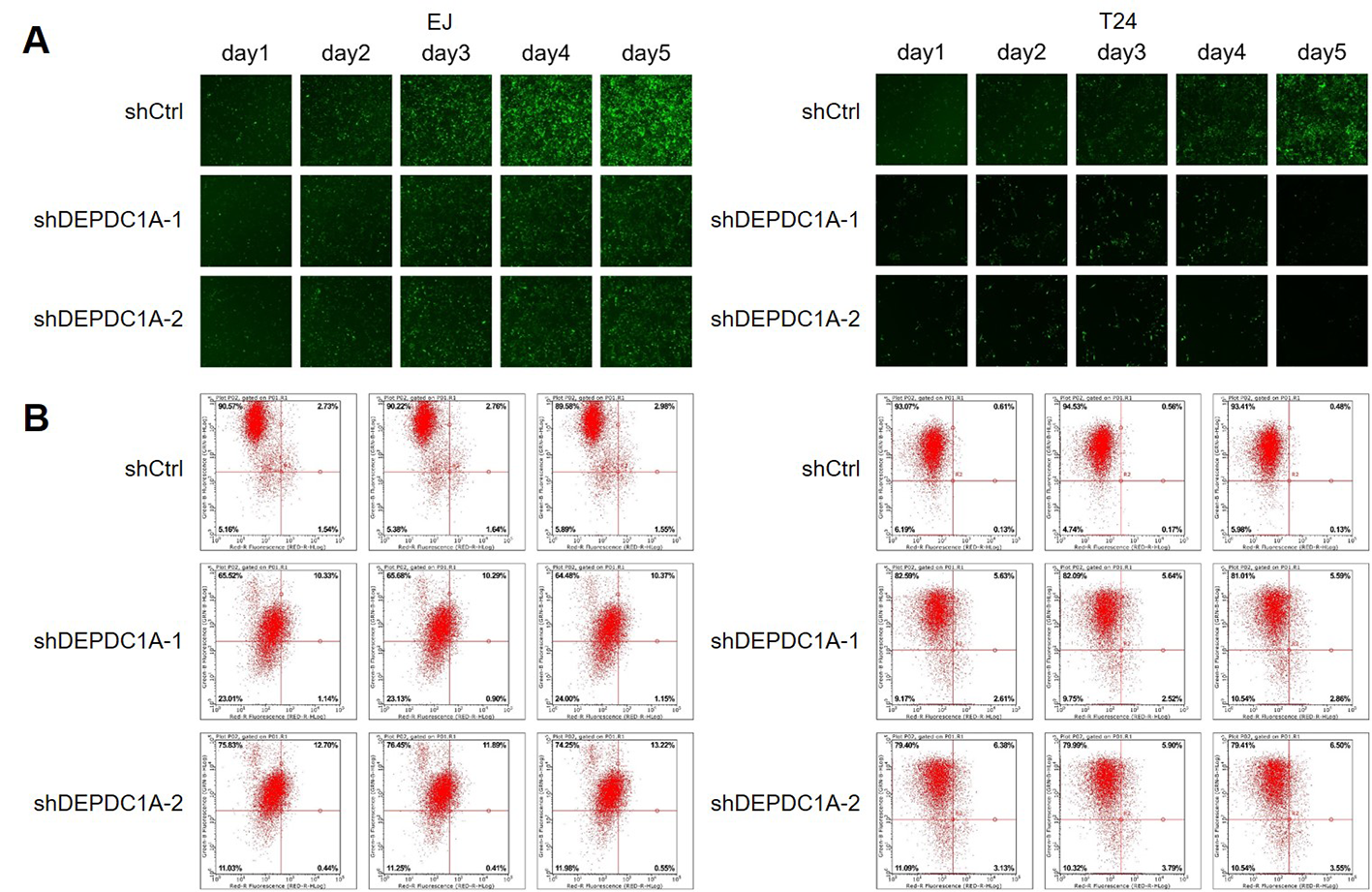

Supplement: Supplementary file 11 — Figure S6 [file 41419_2021_3947_MOESM11_ESM.tif]

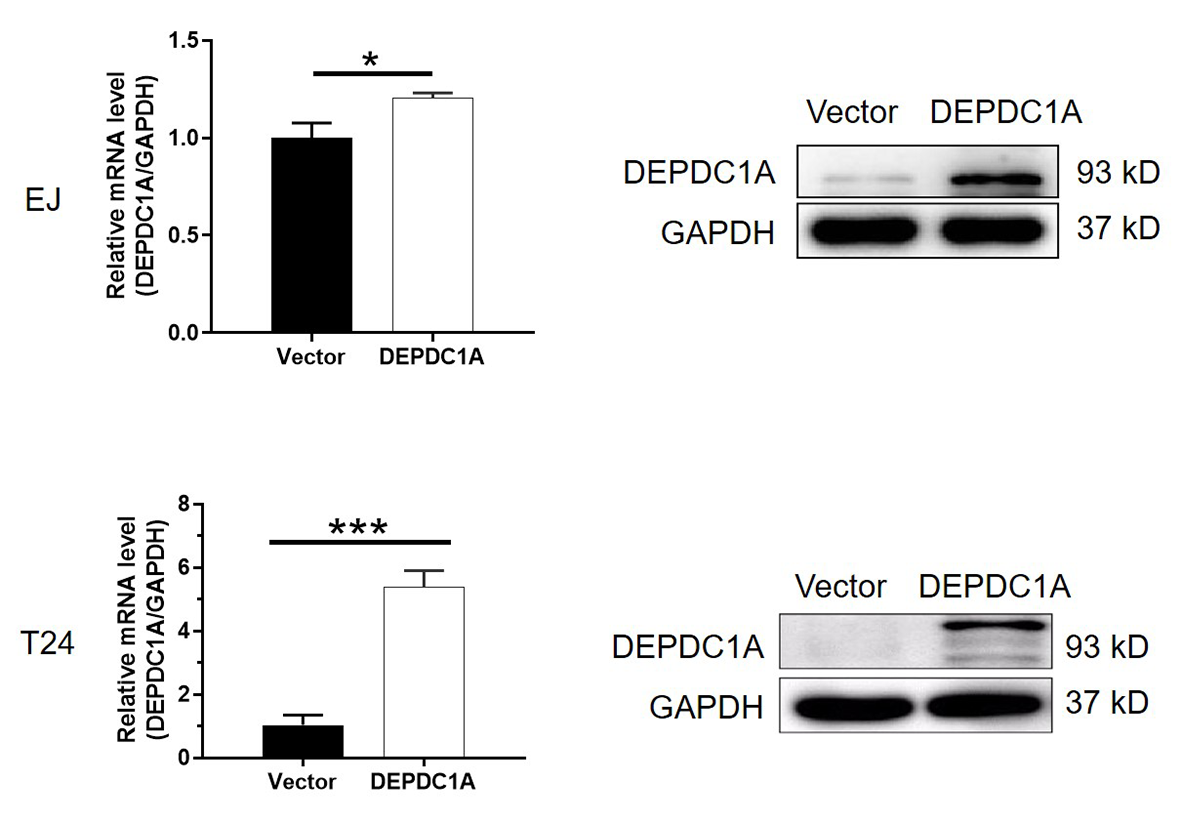

Supplement: Supplementary file 12 — Figure S7 [file 41419_2021_3947_MOESM12_ESM.tif]

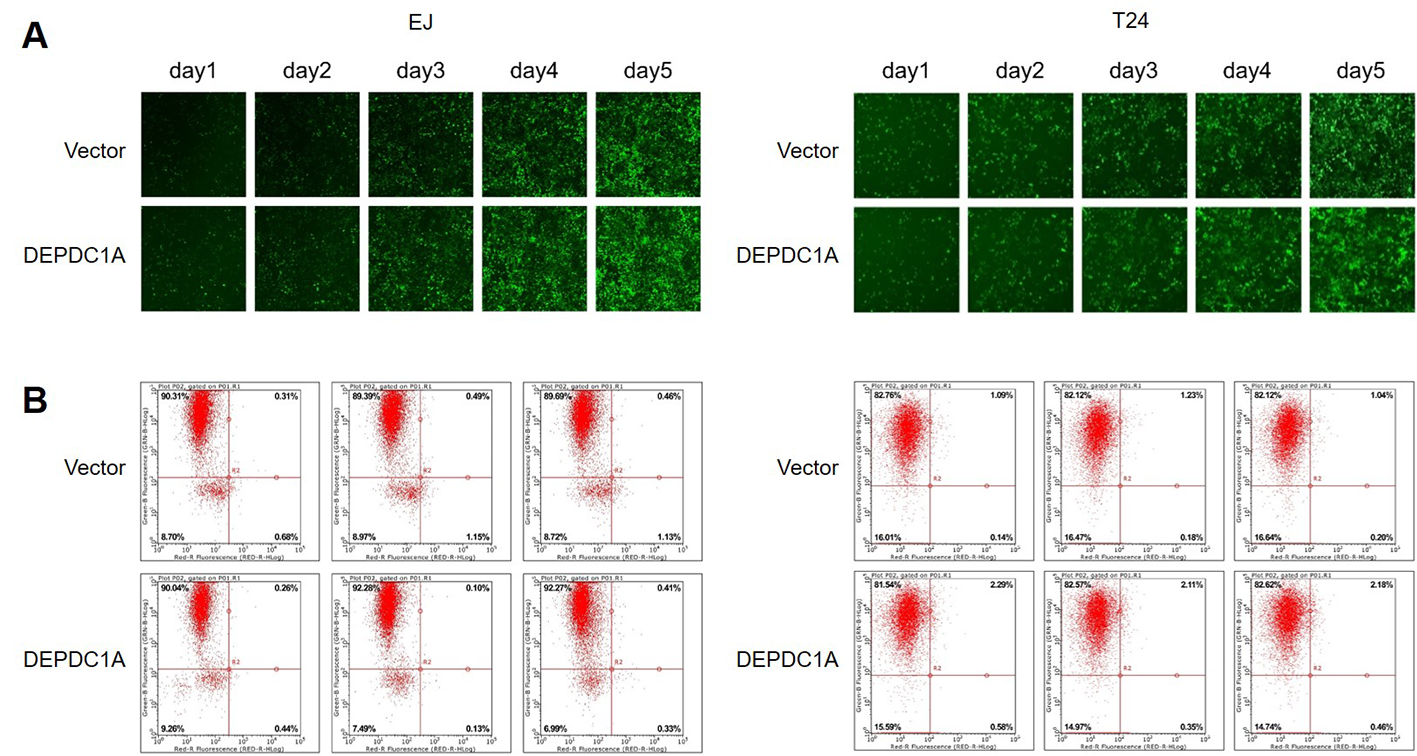

Supplement: Supplementary file 13 — Figure S8 [file 41419_2021_3947_MOESM13_ESM.tif]

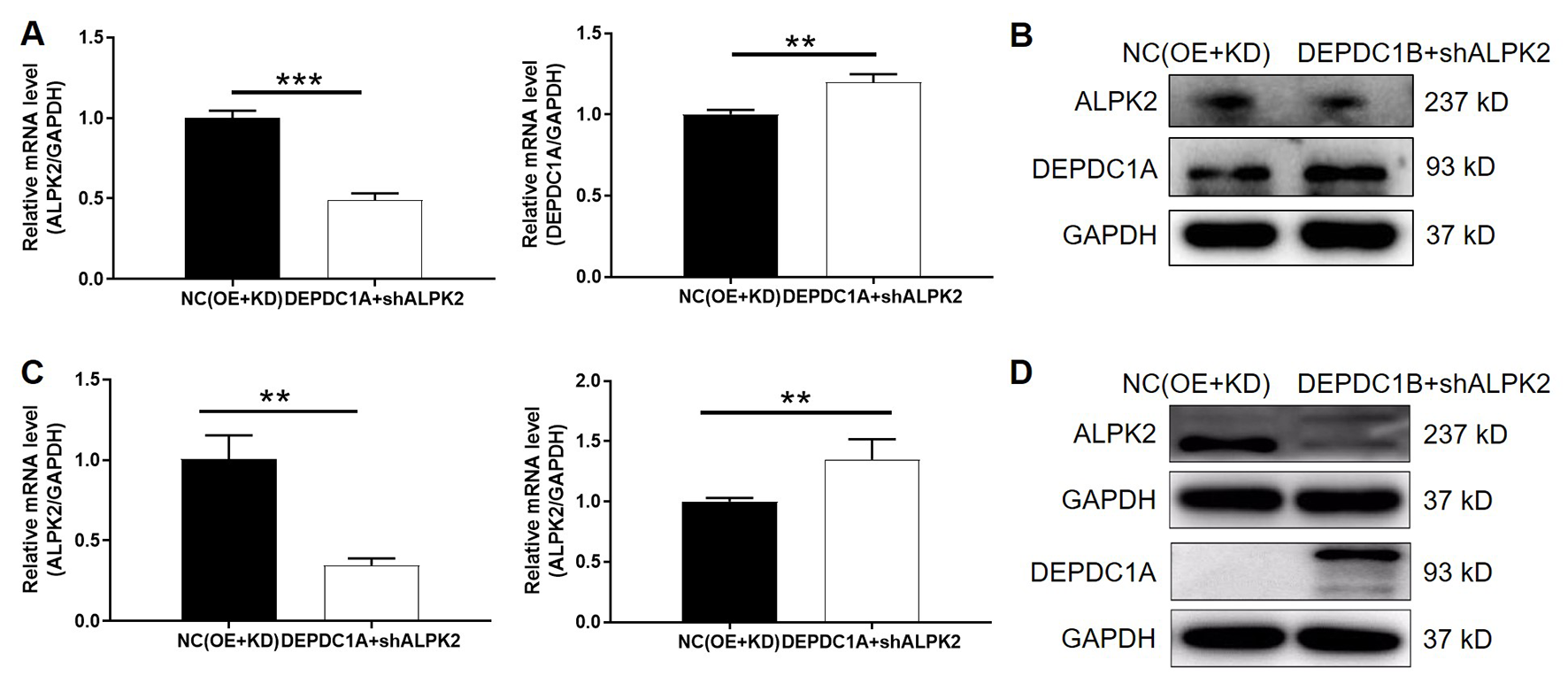

Supplement: Supplementary file 14 — Figure S9 [file 41419_2021_3947_MOESM14_ESM.tif]
